# Antihuman Immunodeficiency Virus Type 1 (HIV-1) Activity of Rare Earth Metal Complexes of 4-Hydroxycoumarins in Cell Culture

**DOI:** 10.1155/BCA/2006/71938

**Published:** 2006-03-02

**Authors:** Ilia Manolov, Sevda Raleva, Petya Genova, Alexey Savov, Liliana Froloshka, Daniela Dundarova, Radka Argirova

**Affiliations:** ^1^Department of Organic Chemistry, Faculty of Pharmacy, Medical University, 2 Dunav Street, 1000 Sofia, Bulgaria; ^2^Department of Virology, National Center of Infectious and Parasitic Diseases, 44A Stoletov Street, 1233 Sofia, Bulgaria; ^3^Laboratory of Molecular Pathology, Medical University, 2 Zdrave Street, 1606 Sofia, Bulgaria

## Abstract

The cerium Ce(III), lanthanum La(III), and neodymium Nd(III) complexes with 4-hydroxy-3-(3-oxo-1-phenylbutyl)-2H-1-benzopyran-2-one (warfarin) (**W**) and 3,3′-benzylidenebis[4-hydroxycoumarin] (**1**) were synthesized and studied for the first time for cytotoxicity (on MT-2 cells) and as anti-HIV agents under acute and chronic infection. The complexes were characterized by different physicochemical methods: mass spectrometry, ^1^H NMR, ^13^C NMR, and IR spectroscopy. The spectra of the complexes were interpreted on the basis of comparison with the spectrum of the free ligands. Anti-HIV effect of the complexes/ligands was measured in MT-2 cells by microtiter infection assay. Detection of endogenous reverse transcriptase (RT) activity and RT processivity by PCR indicative for proviral DNA synthesis demonstrated that anti-HIV activity has not been linked to early stages of viral replication. No effect on late steps of viral replication has been found using cells chronically producing HIV-1_LAI_ virus. La(**W**) demonstrated anti-HIV activity (IC50=21.4 *μ*M) close to maximal nontoxic concentration. Nd(**W**), Ce(**1**), and Nd(**1**) demonstrated limited anti-HIV potency, so none of the complexes seems appropriate to be used in clinic. Further targeting of HIV-1 inhibition by La(**W**) is under progress.

## INTRODUCTION

The need for new HIV inhibitors especially directed to so far unexploited primary targets (integrase, RNase H, viral assembly, etc) is considered to be promising for the development of anti-HIV drugs. Coumarins and bicoumarins are widely spread in nature [1, 2]. Their biological properties are also well known and include anticoagulant, antiproliferative, antimicrobial, spasmolytic, antitumor, antioxidant, and anti-HIV effect reported recently [3].

A large number of structurally novel coumarin derivatives have been reported to demonstrate anti-HIV activity in vitro and in vivo [4, 5]. Plant-derived and semisynthetic calanolide compound exhibited in vitro activity against HIV-1 and human cytomegalovirus [6] and has been proven to be a naturally occurring nonnucleoside reverse transcriptase (RT) inhibitor [1]. Tipravirin, a novel HIV-1 protease inhibitor, has been developed from a nonpeptidic coumarin template [7]. Mazumder et al [8], investigating the effect of several HIV-1 protease inhibitors containing 4-hydroxycoumarin (4-hc) residues, have found antiviral, antiprotease and anti-integrase activities. It has been reported that coumarin and a number of its 4-hc and 7-hc derivatives, including warfarin (**W**), display cytotoxic effect against tumour cell growth and metastases dissemination [9, 10].

Unfortunately, little is known about the complexing ability of lanthanides—cerium Ce(III), lanthanum La(III), and neodymium Nd(III)—with coumarins. Earlier it has been shown that interesting metalorganic compounds of mendiaxon, warfarin, coumachlor, and niffcoumar with lanthanides displayed antitumor activity against P3HR1, K-562, and THP-1 cell lines [11–15]. The complexes had stronger cell proliferation-inhibiting effects compared to the inorganic salts. One could expect that lanthanide complexes with selected active coumarin ligands should retain or even improve their biological activity similarly to other lanthanide complexes with hc derivatives.

Here we present the coordination ability of 4-hydroxy-3-(3-oxo-1-phenylbutyl)-2H-1-benzopyran-2-one (warfarin) (**W**) and 3,3′-benzylidenebis[4-hydroxycoumarin] (**1**) in complexation reaction with Ce, La, and Nd. An increased anti-HIV activity of La(**W**) (IC50=21.4 *μ*M) as compared to (**W**) (7.1% inhibition at maximal nontoxic concentration—MNC) has been demonstrated. Further study has shown limited anti-HIV potency of Nd(**W**), Ce(**1**), and Nd(**1**), so none of the complexes seems appropriate to enter clinical trials. Studies indicated clearly that HIV-1 RT and protease for all complexes were not the targets of antiviral activity.

## EXPERIMENTAL

### Source substances for synthesis

Merck products p.a. Ce(NO_3_)_3_ · 6H_2_O, La(NO_3_)_3_ · 6H_2_O, Nd(NO_3_)_3_ · 6H_2_O. 4-Hydroxy-3-(3-oxo-1-phenylbutyl)-2H-1-benzopyran-2-one (**W**) and 3,3′-benzylidenebis[4-hydroxycoumarin] (C_25_H_16_O_6_) **(1)** were synthesized by us and were used to prepare the solutions.

Ln(III) *complexes of (**W**) * were synthesized using warfarin sodium salt. The synthesis and analysis of the complexes of (**W**) were described previously [11].

*The complexes of lanthanides(III) with **(1)*** were synthesized by reaction of lanthanide(III) salts and the ligand in amounts equal to metal: ligand molar ratio of 1 : 2. The complexes were prepared by adding an aqueous solution of lanthanide(III) salts to an aqueous solution of the ligand subsequently raising the pH of the mixture gradually to ca 5.0 by adding dilute solution of sodium hydroxide. The reaction mixture was stirred with an electromagnetic stirrer at 25°C for an hour. The precipitate formed at the moment of solution mixing was filtered, washed several times with water, and dried in a dessicator to constant weight. The complexes were insoluble in water, slightly soluble in methanol and ethanol, and well soluble in DMSO.

### Analytical and spectroscopic measurements

The elemental analyses for C, H, Ln, and H_2_O were performed according to standard microanalytical procedures. The IR spectra (Nujol and KBr) were recorded on IR-spectrometer FTIR-8101M Shimadzu (3800–400 cm^−1^) and on IR-spectrometer Perkin-Elmer GX Auto image system (700–200 cm^−1^). The ^1^H NMR spectra were recorded at room temperature on Brucker WP 250 (250 MHz) spectrometer in DMSO-d_6_. The ^13^C NMR spectra were recorded at ambient temperature on Brucker 250 WM (62.9 MHz) spectrometer in DMSO-d_6_. Chemical shifts are given in ppm downfield from TMS. The mass spectra were recorded on a Jeol JMS D 300 double-focusing mass spectrometer coupled to a JMA 2000 data system. The compounds were introduced by direct inlet probe, heated from 50°C to 400°C at a rate of 100°C/min. The ionization current was 300 mA, the accelerating voltage 3 kV, and the chamber temperature 150°C.

### Cells, viruses, and assays

#### Cell lines

The following cell lines were used: H9/HTLV III B—chronically infected with HIV-1_LAI_ cells, kindly provided by Professor R. Gallo, and MT-2 uninfected human lymphoblastoid cells, HTLV-I transformed. The cells were maintained in RPMI 1640 plus 10% FCS.

#### HIV-1 source

As a source of HIV-1, the supernatant of H9/HTLV III B cell line was used. The supernatants were collected and centrifuged to remove the cells, and virus stocks were prepared with known p24 antigen content, RT activity and infectivity. MT-2 cells were infected with HIV-1 (multiplicity of infection ≥ 1) in suspension (37°C/5% CO_2_) and then grown parallely with uninfected ones for 72 hours.

#### Cytotoxicity and anti-HIV assays

The following parameters were studied: cytotoxic concentration 50—CC50 (concentration preventing death of 50% of MT-2 cells), maximal nontoxic concentration—MNC (the highest concentration causing no cytotoxicity), and inhibitory concentration 50–IC50 (concentration inhibiting by 50% the viral replication). CC50 and MNC were detected by neutral red uptake assay [16]. IC50 was studied on MT-2 cells by microtiter infection assay using neutral red uptake [16]. Briefly, the experimental procedure under conditions of acute infection was performed in 96-well microplates in the following sequence:

- 100 *μ*L MT-2 cells (4–5 × 10 ^4^ cells);
- 50 *μ*L HIV-1 (undiluted or in the appropriate dilution to obtain multiplicity of infection ≥ 1); contact (2 hours at 37°C/5% CO_2_); when CC50 and MNC were detected, growth medium instead of virus has been added;
- 50 *μ*L sample of substance tested starting from 4× MNC (because this volume is 1/4 of the whole volume in the well) and 2- or 10-fold dilutions in growth medium.

The cells were incubated for 72 hours and then 100 *μ*L of them were removed to another 96-well plate previously treated for an hour with 50 *μ*g/mL poly-L-lysine (PLL) in volume 100 *μ*L. A solution of 100 *μ*L per well of neutral red in growth medium (0.016%) was added and the cells were incubated for 2 hours at 37°C/5% CO_2_. Neutral red dye was extracted by acidified alcohol (50% ethanol in 1% acetic acid) and measured colorimetrically at 540 nm. Each plate contained 8 cell control wells (no virus, no substance) for all kinds of experiments. When IC50 was detected, a row of 8 viral controls (plus virus, no substance) and rows of 8 experimental wells (plus virus, different dilutions of the substance) were performed. The values of optical density at 540 nm (OD540) were averaged for each row and the mean values of experimental and control rows were compared. As far as the ligands and their complexes were DMSO soluble, the appropriate controls of DMSO and DMSO + HIV-1 in growth medium were always considered.

The effect of the compounds was also studied under conditions of chronic infection using productively HIV-infected cells (H9/HTLV III B). The production of infectious virions was measured again using the cytopathic effect of supernatants on MT-2 cells as described above.

#### Detection of RT activity

*Endogenous* RT activity of supernatants of HIV-1 infected MT-2 cells treated/untreated with complexes and ligands was tested by HS-Lenti Kit-RT assay (Cavidi, Sweden). The kit contained recombinant RT (rRT) as a standard which made possible quantitation of the RT in the sample. The method is nonradioactive and colorimetrically detects RT activity product (DNA) at 405 nm (OD405) [3].

#### RT processivity by polymerase chain reactions

The idea was to demonstrate how far the proviral DNA synthesis has progressed and whether mRNA splicing has occurred within HIV-infected cells. (PCRs) were performed using primers M661 and M667 detecting 200 bp product considered expressive for double-stranded (ds) proviral DNA [17]. DNA for PCR was extracted from MT-2 (negative control), HIV-1 infected MT-2 untreated cells (positive control)/cells treated with IC50 or MNC (when IC50 has not been reached) and on 4 and 24 hours after HIV-1 infection. The cells were collected in lysis buffer (pH = 8.3, TRIS-HCl—10 mM, KCl—50 mM, MgCl_2_—2.5 mM, NP40—0.5%, and between 0.5% and 20% [17]. Nucleol-izing buffer (500 *μ*L) containing 30 *μ*L proteinase K and 25 *μ*L 10% SDS was added to the cell lysate. DNA was extracted by phenol-chloroform-isoamyl alcohol method. The detection of amplified product was done after 35 cycles in PCR machine and then visualized in 1.5% agarose gel.

## RESULTS AND DISCUSSION

### Characterization of Ln(III) complexes of (W)

The compositions of the complexes of (**W**) were confirmed by elemental analysis, DTA and TGA, and mass-spectral analysis. The binding mode of (**W**) was further elucidated by IR and ^1^H NMR spectra of the complexes as compared with this of the free ligand. Some of these data were presented by us previously [11].

### Characterization of Ln(III) complexes of (1)

The elemental analysis data of the Ln(III) complexes obtained are in agreement with the formula Ln(L)(OH) · nH_2_O, where L = C_25_H_14_O_6_^2−^. The results of the elemental analyses were within ±0.4% of the theoretical values. The suggested formula was further confirmed by mass-spectral fragmentation analysis. As seen in Table 1, the first peak in the Ln(III) complexes spectra (although with low intensity) corresponds to the mass weight of the complex formation and the second one to that of the ligand. The results thus obtained are in agreement with metal:ligand ratio, 1 : 1.

The binding mode of **(1)** to Ln(III) was further elucidated by analysis of the IR spectra of **(1)** and the complex formation. A broadband characteristic for *ν*(O−H) of coordinated water was observed in the spectra of the complexes in the range of 3500–3450 cm^−1^. The most notable change in the ligand spectral features when coordinated to Ln(III) is the observed C=O red shift. The *ν*(C=O) band at 1660 cm^−1^ in the ligand spectrum exhibited a red shift of 40 cm^−1^ in the spectra of the complexes. This finding may be taken as evidence for participation of the C=O group in coordination to the metal ion. Further, a comparison between the ligand and complex IR spectra revealed that the absorption bands associated with the stretching *ν*(O−H) of the phenolic groups (observed at 3074 cm^−1^ and 3032 cm^−1^ in the free ligand) disappeared in the Ln(III) complex spectra, indicating a loss of phenolic protons on complexation and thus forming a metal-oxygen bonds. The *δ*(COH) IP modes, which appeared at 1345 cm^−1^ and 1336 cm^−1^ in the spectrum of the ligand, were not observed in the spectra of the complexes with Ln(III) and thus supported the suggestion that the ligand coordinates to the metal through its deprotonated form, L^2−^. The Ln(III) complex spectra showed new bands, in comparison with that of the free ligand, at 570 cm^−1^ and 410 cm^−1^, and they were assigned to metal-oxygen stretching vibrations, in agreement with literature.

The Ln(III) complexes and (**1**) were further studied by their ^1^H and ^13^C NMR spectra. The changes of chemical shifts of the ^1^H NMR spectra were observed in the complexes (Table 2) and they were attributed to coordination of the ligand to Ln(III). The chemical shifts of the protons vary in the lanthanide complexes, because of the shift properties of these metals.

Due to electron transfer from the hydroxyl and carbonyl oxygen atoms to Ln(III), chemical shifts to lower ppm were observed for the neighboring C-4 and C-2 carbon atoms of the complexes and they confirmed the expected coordination of the ligand through both deprotonated hydroxyl and carbonyl oxygen atoms (Table 3). The other carbon atoms were only slightly affected from the coordination of the metal. On the basis of the results thus obtained, it was suggested that the ligand acts as a tetradentate one in the Ln(III) complex formation.

### Cytotoxicity and anti-HIV assays

In Table 4, the cytotoxicity (expressed as CC50) and MNCs of both ligands and their lanthanide complexes are shown. As seen, the Nd complex of **(1)** demonstrated at least 5 times lower cytotoxicity compared to the other lanthanides and the ligand **(1)** itself. MNCs of the ligands differed in between the complexes but were maximally nontoxic at 10–50 times higher concentrations for Nd**(1)** and Ce**(1)** compared to La(1) and the ligand **(1)**. The ligand (**W**) and its complexes demonstrated both equal cytotoxicity and equal MNCs. As far as intensive research is often being done to reduce the toxicity of a compound, we demonstrate here that the complexation with rare earth metals possibly leads to increase of MNCs of some derivatives.

Dose-effect dependence of ligands **(1)** and (**W**) and their lanthanide complexes in HIV-1 infected MT-2 cells according to microtiter infection assay [16] are listed in Table 5.

The results display the effect on HIV replication of all ligands/compounds in microtiter infection assay, starting from MNC. As seen, the inhibitory effects in MT-2/HIV-1 system at or under the MNC values are very low for all but one of the compounds under study—La(**W**)—58.4%. It is the only derivative where IC50 could be reached—21.4 *μ*M. All other compounds exhibit low antiviral potency according to this assay.

Looking for the target of antiviral activity especially of La(**W**) as well as of (**W**) and Nd(**W**), Ce(**1**), and Nd(**1**), we detected the endogenous RT activity in the supernatants of HIV-1/MT-2 infected cells and compared it to the results from microtiter infection assay at similar conditions—treatment for 72 h with MNC. The results are demonstrated in Table 6. Data indicate a discrepancy in anti-HIV effect measured by microtiter infection assay and endogenous RT assay. This lack of correspondence is especially well expressed for the only active compound—La(**W**). The results show that the demonstrated anti-HIV activity cannot be explained by inhibition of RT activity—that is, RT is not the target of anti-HIV action. Therefore, another target to explain the effect of La(**W**) on HIV replication should be looked for.

**Figure 1:**
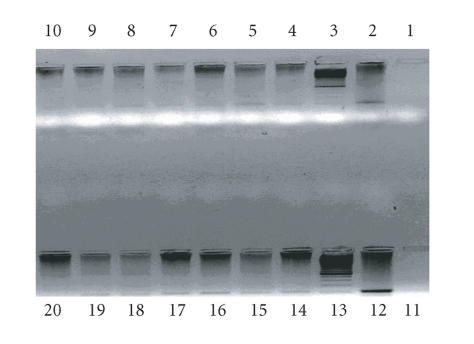
PCR representing HIV ds DNA synthesis after treatment with ligands **(1)** and (**W**) and the complexes with anti-HIV activity in microtiter infection assay (see Table 6). 35 PCR cycles. Amplification conditions: 93°C 30 s, 57°C 90 s, 72°C 30 s. 1.5% agarose gel.

Additional evidence that RT processivity has not been influenced by (**1**) and (**W**) and their complexes having shown some anti-HIV effect were provided by PCR. Figure 1 shows that HIV double-stranded (ds) proviral DNA synthesis was not impaired by the complexes. One can see that 4 h after treatment of HIV-infected MT-2 cells ds proviral DNA was already observed in both treated (lanes 4, 5, 6, 7, 8, 9) and untreated HIV-infected cells (lane 2), while MT-2 uninfected cells displayed no ds DNA (lane 10). The same results but with more intense signal were registered 24 h after treatment of HIV-infected MT-2 cells with (**1**) and (**W**) compounds (lanes 14, 15, 16, 17, 18, 19). Therefore, the results confirm that neither the ligands nor their rare metal ions complexes have an influence on the reverse transcription. So, once again, the discrepancy in anti-HIV activity measured by microtiter infection assay and endogenous RT detection is clearly demonstrated. The conclusion is that the active La(**W**) and the other less potent anti-HIV agents have no impact on the early steps of HIV replication.

To address further the target of HIV inhibition especially by La(**W**), a study of this complex for effect on late stages of HIV-1 life cycle (protease, budding, assembly) was carried out. To do this, we treated H9/HTLV III B cells—chronically producing HIV-1—with La(**W**) at 3 concentrations, starting from MNC. Also, 10× dilution of Indinavir was used as a reference for antiprotease effect. Table 7 represents the results for the effect of La(**W**) in 3 concentrations on late stages of HIV replication.

As seen, H9/HTLV III B cells produced almost equivalent RT activity when treated with La(**W**) in 3 concentrations. Additionally, the number of infectious virions as measured by endpoint dilution analysis on MT-2 cells was equivalent (only OD540 values for undiluted samples are shown—Table 7, column (c)). The data in Table 7 clearly demonstrate no effect of La(**W**) on late stages of HIV-1 replication in cell culture. We especially accentuate that the late stages in this study include at least three steps—protease activity, budding, and assembly of HIV-1.

The fact that no effect on RT and protease (expressing early and late stages of HIV-1 replication) was registered; one can suggest that RT and protease are not targets for the antiviral activity of La(**W**). HIV-1 replication is too complicated and a number of targets and steps (except those studied here) should be taken into consideration (attachment, coreceptor binding, fusion, RNase H-activity, integration, inhibition of glycosylation or sialylation, etc). Although not especially being a subject of attachment studies, the results of RT assays (Table 6) and RT processivity (Figure 1) clearly indicate no effect of the complexes on the earliest steps of replication. Research is now in progress to look for putative inhibitory effect of La(**W**) first on integration and then further to specify the mechanism of its action.

Here we show for the first time both complexation of rare earth metal ions with selected coumarins and study of their effect on HIV replication in cell culture. Although the complexes are of low potency against HIV-1 and should not enter clinical trials, the experience with them shows once more that the cytotoxicity could be reduced and the antiviral effect—highly expressed through complexation reactions. Lack of protease and RT activity has been recently reported for a number of HIV-1 integrase inhibitors [18] and on the contrary, antiviral agents inhibiting both integrase and protease have also been published [8]. The unique and variable activities of plant-derived and semisynthetic coumarins, as well as novel approaches for synthesis of potent and less toxic derivatives could facilitate generation of new lead compounds for chemotherapeutic intervention. The lanthanide complexes with selected coumarins could offer such an opportunity.

## Figures and Tables

**Table 1:** Data of the mass spectra of the complexes of (**1**).

Ligand/complexes	M/z	(%)

H_2_L=C_25_H_16_O_6_	412	8
	249	100
	221	17
	162	20
	120	37
Ce(L)(OH) · 2H_2_O	586	8
	410	35
	305	98
	176	100
La(L)(OH) · H_2_O	586	8
	410	35
	305	100
	176	100
Nd(L)(OH) · H_2_O	589	2
	410	1
	307	68
	176	100

**Table 2:**
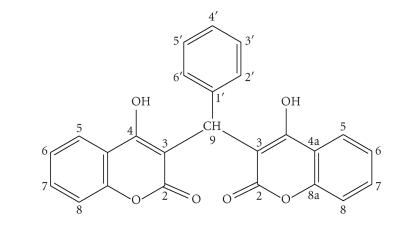
^1^H NMR spectra of the complexes of (**1**).

Compound	H_5_–H_8_^a^	*δ* (ppm) H_9_^a^	H_2′_–H_6′_^a^

H_2_L=C_25_H_16_O_6_	7.11–7.39	6.37	7.56–7.92
Ce(L)(OH) · 2H_2_O	7.05–7.24	6.25	7.45–8.18
La(L)(OH) · H_2_O	9.98–7.26	6.27	7.37–7.83
Nd(L)(OH) · H_2_O	7.47–7.60	6.64	7.88–8.16

**Table 3:** ^13^C NMR spectra of the complexes of (**1**).

Atom	*δ* (ppm)			

	H_2_L	Ce(L)(OH) · 2H_2_O	La(L)(OH) · H_2_O	Nd(L)(OH) · H_2_O

C-2	165.3	167.8	164.6	167.7
C-4	164.9	164.7	152.7	164.6
C-8a	152.2	152.5	152.5	152.5
C-1′	139.9	142.3	142.4	142.3
C-7	131.9	130.9	130.9	130.9
C-3′	128.1	127.7	127.7	127.6
C-5′	128.1	127.7	127.7	127.6
C-4′	126.7	126.6	126.6	126.6
C-6′	125.6	124.8	124.8	124.8
C-2′	125.6	124.8	124.8	124.8
C-5	123.9	124.1	124.1	124.1
C-6	123.8	122.9	122.9	122.9
C-4a	117.9	119.9	119.9	119.9
C-8	115.9	115.4	115.4	115.4
C-3	104.1	103.4	103.4	103.4
C-9	35.9	36.5	37.5	36.5

**Table 4:** Cytotoxicity as CC50 and MNCs of (**1**) and (**W**) and their Ce, La, and Nd complexes studied by neutral red uptake assay [16].

Ligand	Ligand		Ce		La		Nd	
	only		complex		complex		complex	

	CC50	MNC	CC50	MNC	CC50	MNC	CC50	MNC
	*μ*M	*μ*M	*μ*M	*μ*M	*μ*M	*μ*M	*μ*M	*μ*M

**(1)**	25	0,25	25	12,5	25	0,25	125	2.5
(**W**)	250	25	250	25	250	25	250	25

**Table 5:** Dose-effect dependence of ligands (**1**) and (**W**) and their lanthanide complexes in HIV-1_LAI_ infected MT-2 cells (72 h incubation) according to microtiter infection assay [16]. The concentrations tested start with MNC for each ligand/compound (see Table 4).

Ligand/	Concentration	Inhibition
compound	(*μ*M)	(%)

(**W**)	25	7,1
	2.5	0
Ce(**W**)	25	0
La(**W**)	25	58,4
	2.5	2,0
	0,25	0
Nd(**W**)	25	6,9
	2.5	0
**(1)**	0,25	0
Ce**(1)**	12,5	11,5
	2.5	8,7
	0,25	0
La**(1)**	0,25	0
Nd**(1)**	2.5	10,4
	0,25	0

**Table 6:** Comparison of inhibition (%) of HIV-1 replication in MT-2 cells by microtiter infection assay and endogenous RT assay by some lanthanide complexes of (**1**) and (**W**) at MNC (72 h incubation).

Complex/	MNC	HIV inhibition (%)	HIV inhibition (%)
ligand	(*μ*M)	*Microtiter*	*Endogenous*
		*infection assay* *	*RT activity* *

Ce(**1**)	12.5	12.2	∼ 1
Nd(**1**)	2.5	10.4	∼ 1
La(**W**)	25	58.4	∼ 1
Nd(**W**)	25	6.9	∼ 1
(**W**)	25	7.1	∼ 1

*Data averaged after at least 3 experiments.

**Table 7:** Data obtained after treatment of H9/HTLV III B cells with different concentrations of La(**W**) to target late stages of HIV-1 replication (protease, budding, assembly).

			RT	Microtiter
		Concentration	activity of	infection
Compound	Cells	(*μ*M)	SNs^1^	assay^2^
			*OD405*	*OD540*
		(a)	(b)	(c)

H9/HTLV III B		25	2.980	1.530
treated with		2.5	2.987	1.525
La(**W**)		0.25	2.926	1.520

	SN from	—	0.244	1.501
	MT-2 cells			

	SN from	—	2.859	0.434
	MT-2/HIV-1			

	SN from	—	2.872	1.539
	H9/HTLV			
	III B			

H9/HTLV III B		50	0.903	0.375	
treated				
with				
Indinavir				

		5	1.154	0.520
		0.5	1.400	0.633
		0.05	1.454	0.739

^1^SN–supernatant.

^2^H9/HTLV III B were treated for 72 hours with La(**W**) in designated concentrations (a) and RT activity in supernatants was measured (b). Thereafter, MT-2 cells were infected with 50 *μ*L of undiluted supernatants of H9/HTLV III B cells from (b) and microtiter infection assay was carried out as described (c)—see *Experimental*.
